# Prevalence of Polycystic Ovarian Syndrome Among Young College Girls: A Cross-Sectional Study

**DOI:** 10.7759/cureus.92623

**Published:** 2025-09-18

**Authors:** S Niranjani, Krishnan Prema, Golda Sahayarani, Dinesh M, Carline K

**Affiliations:** 1 Community Health Nursing, Shri Sathyasai College of Nursing, Sri Balaji Vidyapeeth, Chennai, IND; 2 Child Health Nursing, Shri Sathyasai College of Nursing, Sri Balaji Vidyapeeth, Chennai, IND; 3 Medical Surgical Nursing, Shri Sathyasai College of Nursing, Sri Balaji Vidyapeeth, Chennai, IND

**Keywords:** cross-sectional study, endocrine disorder, metabolic disorder, polycystic ovarian syndrome, prevalence

## Abstract

Introduction

Polycystic ovarian syndrome (PCOS) is one of the most common endocrine disorders among young girls, often resulting in hormonal imbalances, menstrual irregularities, and metabolic disturbances. It is linked to long-term health risks such as obesity, insulin resistance, and cardiovascular disease. Early identification and intervention are critical for managing symptoms and preventing complications. This study was undertaken to determine the prevalence of PCOS among young girls by assessing multiple diagnostic markers.

Materials and methods

Formal permission was obtained from the deans and principals of the selected institutions before initiating the study. The purpose of the research was explained to all participants, and written informed consent was secured. Demographic information was collected using a structured questionnaire. A total of 824 students were initially screened for PCOS using standardized tools based on the Rotterdam diagnostic criteria. Students identified as potential cases subsequently underwent ultrasonography, physiological measurements, and biochemical investigations to confirm the diagnosis.

Results

Totally 824 students were screened for PCOS, and 232 were identified as potential cases using the Rotterdam-based screening tools. These participants underwent further diagnostic evaluations, including ultrasonography, physiological measurements, and biochemical testing. Among them, 161 students were confirmed to have PCOS, yielding a prevalence rate of 19.5% in the study population (n = 824).

Conclusion

This study highlights a considerable prevalence of PCOS among young girls when assessed using comprehensive diagnostic methods. Early screening based on the Rotterdam criteria was effective in identifying potential cases for further evaluation. The findings emphasize the need for routine screening and timely interventions in young females to facilitate early diagnosis and reduce the risk of long-term complications.

## Introduction

Polycystic ovarian syndrome (PCOS) is recognized as a common endocrine disorder affecting females of reproductive age, with its onset often observed during adolescence or early adulthood [1]. It is characterized by a combination of clinical, biochemical, and ultrasonographic features. PCOS presents with a spectrum of symptoms, including menstrual irregularities, hyperandrogenism manifesting as hirsutism, acne, and alopecia, and polycystic ovaries [2]. In addition to reproductive concerns, PCOS is associated with systemic complications such as insulin resistance, obesity, dyslipidemia, and an elevated risk of type 2 diabetes and cardiovascular disease [3]. Early identification of PCOS is critical to facilitate timely intervention and prevent long-term complications [4]. However, diagnosis remains challenging due to the variability in clinical presentation and the lack of awareness among adolescents and healthcare providers [5]. The Rotterdam diagnostic criteria, requiring any two of the following three features: oligo/anovulation, clinical or biochemical signs of hyperandrogenism, and polycystic ovaries on ultrasound, are widely accepted for diagnosing PCOS [6].

The prevalence of PCOS worldwide is 9.2% [7]. A prevalence rate of PCOS in Indian women is in the range of 3.7% to 36% [8,9]. The increasing prevalence among adolescents and young adults in India is attributed to changing lifestyle patterns, dietary habits, sedentary behavior, and elevated stress levels [1]. Despite its growing impact, there remains a gap in large-scale, institution-based studies evaluating PCOS prevalence using standardized diagnostic tools. This study was undertaken to determine the prevalence of PCOS among young girls in selected educational institutions using comprehensive screening methods based on the Rotterdam criteria. It also aimed to assess associated physiological and biochemical markers, thereby contributing valuable insights into early detection strategies and underscoring the urgent need for health education, preventive measures, and timely intervention programs.

## Materials and methods

A quantitative research approach was adopted for this study, considering the nature of the problem and the quantitative nature of the variables under investigation. A cross-sectional descriptive design was employed to achieve the study objectives. The participants were young girls aged between 18 and 21 years who were studying at the Hindu Mission College of Nursing, Tagore College of Nursing, Tagore College of Pharmacy, Tagore Allied Health Sciences, Tagore Engineering College (TEC), and the Master of Business Administration program at the TEC in Chennai. Convenience sampling was used to recruit participants and colleges.

The tool used for the study consisted of two major components: a screening tool and a confirmatory tool. The screening tool included demographic information and assessments based on the Rotterdam diagnostic criteria [6], while the confirmatory tool incorporated ultrasonography, physiological measurements, and biochemical parameters. A summary of the study tool is presented in Table 1.

**Table 1:** Components of the study tool PCOS: polycystic ovarian syndrome; NABL: National Accreditation Board for Testing and Calibration Laboratories

Part	Section	Description	Method/scale used	Interpretation/criteria
Part I: Screening tool	Demographic variables	Collected baseline details (age, education, type of family, birth order, socioeconomic status, dietary pattern, medical history).	Self-administered questionnaire	Descriptive baseline information
	Oligomenorrhea	Menstrual history, including age at menarche, cycle length, flow duration, number of cycles per year, and family history of PCOS.	Self-administered questionnaire	Irregular/absent cycles classified as ovulatory dysfunction
	Hyperandrogenism	Ferriman-Gallwey Scale (nine body areas).	Scored 0–4 for each area; higher score = greater hirsutism	Presence of ≥2 features indicates hyperandrogenism
	Hirsutism			
	Acne	Global Acne Grading Scale (forehead, cheeks, nose, chin, chest, upper back).	Severity graded by cumulative score	
	Alopecia	Ludwig Visual Alopecia Scale for female pattern baldness.	Stage I (mild thinning) to Stage III (severe loss)	
Part II: Confirmatory tool	Ultrasonography	Pelvic scan for ovarian morphology.	Pelvic ultrasound	≥20 follicles (2–9 mm) and/or ovarian volume ≥10 ml = positive finding
	Physiological parameters	Anthropometric measurements.	Digital weighing scale, measuring tape	BMI <18.5 underweight, 18.5–24.9 normal, ≥25 overweight/obese
	Biochemical parameters	Serum testosterone analysis.	Blood test in an NABL-accredited lab	Reference range: 15–70 ng/dL

Demographic data were collected through a self-administered questionnaire. Screening was conducted according to the Rotterdam diagnostic criteria, which require at least two of the following: hyperandrogenism, ovulatory dysfunction, and polycystic ovaries. Hyperandrogenism was assessed clinically using the Ludwig Visual Alopecia Scale for female pattern alopecia, the Ferriman-Gallwey Scale for hirsutism, and the Global Acne Grading Scale for acne severity [10-12]. Oligomenorrhea was evaluated through menstrual history.

Confirmatory assessment included ultrasonography, physiological measurements, and biochemical testing. Pelvic ultrasonography was used to detect ovarian morphology, with ≥20 follicles (2-9 mm) or ovarian volume ≥10 ml on either ovary considered positive. Physiological parameters included body weight, height, body mass index (BMI), and waist circumference, measured according to World Health Organization recommendations [13]. Biochemical investigation included total serum testosterone, analyzed in an NABL-accredited laboratory, with a reference range of 15-70 ng/dL.

The reliability of the study tool was ensured through several measures. The screening instruments used, including the Ferriman-Gallwey Scale for hirsutism, the Global Acne Grading Scale for acne severity, and the Ludwig Visual Alopecia Scale for female pattern hair loss, are internationally validated clinical tools with established reliability and consistency in assessing hyperandrogenism features. Menstrual history for identifying oligomenorrhea was obtained through self-reported data, which was cross-verified with participants to minimize recall bias. For the confirmatory assessments, ultrasonography was performed by a qualified radiologist using standardized protocols, ensuring inter-observer reliability. Physiological measurements (weight, height, waist circumference, and BMI) were taken using calibrated instruments and repeated twice to maintain accuracy and reduce measurement error. Biochemical investigations (serum testosterone levels) were conducted in a NABL-accredited laboratory, following strict quality control procedures, which ensured both internal and external reliability of test results. To strengthen reliability further, a pilot study was conducted among a small subset of participants (not included in the main study) to assess clarity, feasibility, and consistency of the screening questionnaire. Necessary modifications were made prior to the final data collection.

Ethical approval was obtained from the Institutional Ethics Committee (IEC), Tagore Medical College and Hospital, Chennai (IEC No: 32/MAR/2021, dated March 18, 2021). Permission was also secured from the deans and principals of the participating institutions. The purpose and procedures of the study were clearly explained to participants, and written informed consent was obtained prior to data collection.

A total of 824 female students were screened for PCOS using the standardized tool that incorporated demographic data, gynecological history, the Ferriman-Gallwey Scale for hirsutism, the Global Acne Grading Scale, and the Ludwig Visual Alopecia Scale. Based on the screening results, 232 students were identified as potential participants and subsequently underwent ultrasonography, physiological measurements, and biochemical investigations.

## Results

Screening outcomes

A total of 824 young girls were screened for PCOS using standardized tools aligned with the Rotterdam diagnostic criteria. Among the clinical indicators, the most common was acne (n = 320, 38.8%), followed by alopecia (n = 298, 36.2%), menstrual irregularity (n = 283, 34.3%), and hirsutism (n = 232, 28.2%). The summary of the screening outcomes is presented in Table 2.

**Table 2:** Distribution of PCOS indicators based on screening tool PCOS: polycystic ovarian syndrome

Condition	Tool used	N (respondents)	Prevalence (%)
Menstrual irregularity	Gynaecological data	283	34.30%
Hirsutism	Modified Ferriman-Gallwey Scale	232	28.20%
Acne	Global Acne Grading Scale	320	38.80%
Alopecia	Ludwig Visual Alopecia Scale	298	36.20%

Confirmatory tool outcomes

In addition to clinical features, diagnostic assessments were performed. Anthropometric evaluation (BMI and waist circumference) identified 200 participants (24.3%) with values outside the normal range. Biochemical testing (serum testosterone) showed elevated levels in 170 participants (20.6%). Ultrasonography revealed polycystic ovarian morphology in 161 participants (19.5%). The summary of the confirmatory tool outcomes is presented in Table 3.

**Table 3:** Distribution of PCOS indicators based on confirmatory tool PCOS: polycystic ovarian syndrome

Marker	N (respondents)	Percentage
BMI and waist circumference	200	24.30%
Ultrasound (USG)	161	19.50%
Testosterone	170	20.60%

Confirmed cases of PCOS

Based on comprehensive assessments, including clinical, ultrasonographic, and biochemical parameters, 161 participants were confirmed to have PCOS, yielding an overall prevalence rate of 19.5%.

## Discussion

This study identified a 19.5% prevalence of PCOS among 824 screened young girls, which is supported by the following systematic review and meta-analysis that reported a pooled PCOS prevalence of 17.74% among Indian adolescents aged 14-19 years, based on the Rotterdam criteria [14]. Similarly, it was found that a prevalence of 17.33% in a rural adolescent cohort, underscoring the widespread nature of PCOS across diverse settings [15].

Clinical manifestations in the present study, including acne (38.8%), alopecia (36.2%), menstrual irregularity (34.3%), and hirsutism (28.2%), are consistent with the following studies. For instance, it was observed that 11.96% PCOS prevalence among adolescents presenting with oligomenorrhea and hirsutism [16]. These findings reaffirm that hyperandrogenic symptoms remain hallmark features in the diagnosis of PCOS. Physiological and biochemical assessments further validated our results. In the present study, elevated BMI and waist circumference were observed in 24.3% of participants, while 20.6% demonstrated increased testosterone levels. These findings are in line with previous research, which reported that 44.5% of women were overweight and 35.3% were obese. Additionally, 41.2% had a waist circumference exceeding 35 inches, and elevated total and free testosterone levels were noted in 69.8% and 51.3% of participants, respectively. These comparisons highlight the consistent association between obesity, central adiposity, and hyperandrogenism across different study populations [17]. In the present study, ultrasonography confirmed polycystic ovarian morphology in 19.5% of participants. This diagnostic tool remains essential, as previous research has reported that polycystic ovarian morphology can be observed in up to 57% of women presenting with anovulation and hirsutism [18]. From a global perspective, a pooled adolescent PCOS prevalence of 9.8% has been reported using the Rotterdam criteria, with the highest rates observed in the South-East Asia region [7]. The findings of our study, therefore, align with both national and international data, while highlighting the growing burden of PCOS in Indian adolescents. The increasing prevalence of PCOS among adolescents is attributed to lifestyle changes, dietary patterns, sedentary behavior, and increased screen time. It was reported a concerning rise in PCOS/polycystic ovarian disease (PCOD) cases among teenagers, emphasizing early menarche and hormonal imbalance as contributory factors [19-21]. Overall, this study highlights the need for early, multi-parameter screening in educational institutions. Addressing PCOS in adolescence can reduce the risk of infertility, metabolic syndrome, and type 2 diabetes later in life. Incorporating structured screening programs within schools and colleges could facilitate timely diagnosis and effective management of PCOS, thereby preventing long-term complications.

This study has certain limitations. First, as a cross-sectional study, causal relationships between risk factors and PCOS could not be established. Second, the study was conducted in a single geographical region, which may limit the generalizability of findings to other populations. Future research with larger, multi-center, and longitudinal designs is recommended to validate these findings and better understand the natural course of PCOS in adolescents.

## Conclusions

This study highlights a notable prevalence of PCOS among adolescent girls, identified through a comprehensive screening approach based on the Rotterdam diagnostic criteria. Clinical manifestations such as acne, alopecia, menstrual irregularities, and hirsutism were common, underscoring the importance of early recognition. Incorporating physiological, biochemical, and imaging parameters strengthened diagnostic accuracy and reinforced the value of school-based screening initiatives. Early detection and timely intervention are essential to mitigate the long-term metabolic, reproductive, and psychological complications associated with PCOS. The findings emphasize the urgent need to raise awareness among adolescents, parents, and educators, while also promoting routine health screenings in educational institutions. Establishing adolescent-friendly health services that address both the physical and emotional aspects of PCOS will further enhance management strategies. Future research should prioritize longitudinal studies to monitor outcomes over time and to evaluate the effectiveness of early interventions in reducing the overall burden of PCOS among adolescents.
